# Impact of the Work Environment on Nurse Outcomes: A Mediation Analysis

**DOI:** 10.1177/01939459241230369

**Published:** 2024-02-11

**Authors:** Caroline Boudreau, Ann Rhéaume

**Affiliations:** 1 School of Nursing, Université de Moncton, Moncton, NB, Canada

**Keywords:** nurses, work environment, COVID, missed nursing care, scope of practice, emotional exhaustion, turnover intentions

## Abstract

**Background:**

The nursing workforce remains in a vulnerable state post pandemic as working conditions are difficult and exacerbated by a global nursing shortage. Identifying factors leading to turnover intentions are thus critical for health care system recovery.

**Purpose:**

The purpose of this study was to examine the impact of nurses’ work environment and the pandemic on missed nursing care, scope of practice, emotional exhaustion, and intent to leave.

**Methods:**

This study was a cross-sectional, self-reporting online survey, sent to hospital-based nurses in a Canadian province (n = 419). Mediation analysis was used to examine both direct and indirect effects of work environment and COVID-19 impact on nurse outcomes (emotional exhaustion and intent to leave) through missed care and scope of practice.

**Results:**

The results showed that 73% of nurses were considering leaving the profession. Several direct and indirect pathways predicted emotional exhaustion and intent to leave. A better work environment was related to both decreased emotional exhaustion and intent to leave. Nurses’ scope of practice partially mediated the relationship between work environment and intent to leave. On the other hand, missed care did not mediate emotional exhaustion or intent to leave.

**Conclusions:**

While considering the global nursing shortage, it is imperative to implement strategies to promote nurses’ well-being and their retention within the health care system.

Nurses’ work environment is one of the greatest influences on the quality of care provided to patients and has a major impact on nurses’ well-being.^1^ Unfortunately, the hospital work environment continues to be problematic, with limited available resources and increased job demands, despite the wealth of research available and ensuing initiatives. Furthermore, nurses’ work environment is currently being impacted by the global nursing workforce shortage and recent pandemic. According to the World Health Organization,^2^ there is a shortage of almost 6 million health care professionals worldwide and 1 in 6 nurses will be eligible to retire in the next 10 years. The recent COVID-19 pandemic has also exacerbated the impact of the nursing shortage on the work environment by straining the health care system with unprecedented force and increasing mental health problems in nurses.^3-5^ There is more pressure than ever to provide excellent nursing care, while working with a restricted budget and less resources and staff.^6,7^ Gaining a better understanding of the pathways leading to burnout symptoms and intent to leave is essential to identify key interventions for strategic nursing workforce planning and to promote retention.

## Work Environment and Nursing Practice During the Pandemic

Nurses’ work environment is described as organizational characteristics that promote or restrain nursing practice.^8^ A healthy work environment is satisfying, mentally and physically safe,^1^ and encourages nurses to work to their full potential and provide quality care. Important factors that influence nurses’ work environment are workload and staffing levels, decision-making latitude, job demands and complexity, good leadership, and the ability to participate in unit-based decisions.^9^ Decades of empirical research have demonstrated the strong relationship between work environment traits and various nurse outcomes such as work engagement,^10-13^ job satisfaction,^1,10,11^ burnout,^10-13^ and turnover intentions.^1,10,11,13^ Furthermore, nurses’ work environment is considered an important structural component in Donabedian’s Structure-Process-Outcome model of quality of care.^14^ The pandemic has exacerbated an already challenging work environment by creating new workplace stressors, such as a shortage of personal protective equipment, rapidly changing hospital policies, lack of preparedness for complex patient care, and a fear of disease transmission, especially at the beginning of the pandemic.^15-19^ The pandemic and its successive waves have amplified the work-related demands on nurses, and in return have led to an increased prevalence of burnout symptoms, depression, and post-traumatic stress.^16,17,20,21^

The work environment is one of the most important predictors of missed nursing care.^22-24^ Inadequate staffing and resources, their poor utilization, high workload, and poor communication are all factors that determine the amount of nursing care omitted.^25-27^ Missed nursing care can be described as an error of omission, where the nurse knows that a certain task needs to be done but does not manage to provide care in a timely manner, or at all.^28^ There is a high incidence of missed nursing care in hospital settings across the world, although there are differences between and within countries.^23,29^ The impact of missed nursing care on patients has been studied at length^22,30^ and is seen as an indicator of care delivery processes.^24,25^ Research has shown that the inability to provide care impacts nurses’ well-being by decreasing job satisfaction, increasing burnout symptoms, and consequently increasing turnover intentions.^7,13,24^

Nurses’ scope of practice is defined as the standards of care that are set by regulatory bodies, reflecting the knowledge and skills expected of nurses.^31,32^ In the context of a global nursing shortage, nurses’ practice should be optimized to ensure efficiency and continuity of care.^33,34^ However, many work characteristics have a significant impact on nurses’ scope of practice.^34,35^ For example, lack of resources and staffing inadequacy make it almost impossible for nurses to work using the full extent of their knowledge and skills.^35,36^ Few studies have examined the impact of scope of practice enactment on nurses’ well-being. An exception is a recent correlational study showing a positive relationship between certain care activities (i.e., teaching and emotional support) and job satisfaction.^34^ It can be argued that optimizing nursing practice can improve nurses’ well-being by decreasing stress, encouraging autonomy, increasing satisfaction at work,^35^ as well as increasing retention levels.^33^

## Work Environment and Nurse Outcomes: Emotional Exhaustion and Intent to Leave

While there are different conceptualizations of burnout, emotional exhaustion is considered one of the core dimensions.^37-39^ The prevalence of burnout in nurses is high. Prior to the COVID-19 pandemic, a meta-analysis indicated that around 10% of all nurses suffered high levels of burnout, although these rates differed across regions and specialties.^40^ A recent Canadian study that took place before the pandemic reported that 63% of nurses had symptoms of burnout, while 30% met clinical diagnosis criteria.^41^ Both an unsupportive work environment and missed nursing care are considered predictors of burnout symptoms.^27,37,42^ Emerging research during the pandemic indicated that workplace stressors (i.e., job demands and workload) increased nurses’ burnout levels, while the workforce shortage acted as a catalyst increasing burnout symptoms.^5,17,43-45^ Emotional exhaustion also acts as a mediator between nurses’ participation in hospitals affairs, a characteristic of a good work environment, and nurse outcomes, such as intent to leave.^46^ High rates of burnout also increased the health care system’s expenses, since it causes a decrease of productivity and a rise in absenteeism and turnover intentions.^39,47^

An increased turnover rate impacts morale and teamwork within the staff, consequently increasing workload^48^ and intent to leave.^13,39^ The social and psychological impacts of the pandemic appeared to have increased the turnover rate among nurses,^48^ which is higher 2 years into the pandemic.^4^ According to the International Council of Nurses, 20% of nursing associations globally reported an increased intent to leave among their members.^49^ In the Canadian province of Quebec, 29% of nurses considered quitting their current job and 22% the nursing profession,^16^ while almost 50% of nurses working in Canadian intensive care units during the pandemic considered leaving.^21^

Although there is a growing body of knowledge regarding the work environment and nurse outcomes, there are nonetheless questions remaining about the processes leading to turnover intentions, particularly in the final waves of the pandemic. We argue that both scope of practice and missed care are processes that mediate the relationship between the work environment and nurse outcomes within the current health care context. To our knowledge, these complex relationships have not been studied. With an important shortage of registered nurses, it is of upmost importance to examine the impact of the work environment and to identify strategies ensuring nurses’ well-being and their retention in the profession.

## Purpose

The purpose of this study was to examine the mediating effect of missed care and scope of practice on the relationship between nurses’ work environment and nurse outcomes (emotional exhaustion and turnover intentions) during the last waves of the COVID-19 pandemic. A secondary study objective was to examine the mediating effect of emotional exhaustion on the relationship between work environment and turnover intentions.

### Hypothesized Model

The model used for this study was based on Donabedian’s framework of quality assessment.^14,50^ There are 3 major pillars to Donabedian’s model: structure, process, and outcomes. Structure can be described as the work environment attributes that promote or constrain the nurse’s work in the hospital setting, including the pandemic’s impact. Processes are defined as the care delivered to patients. Finally, outcomes can be described as the impact of the structure and process of patient care on nurse outcomes. In this study, missed nursing care and scope of practice are identified as processes, while emotional exhaustion and intent to leave are identified as outcomes. Several recent studies have used Donabedian’s model to explain the factors influencing the quality of nursing care and its impact on nurse outcomes.^12,24^ We hypothesize that nurses’ work environment and the pandemic will have direct and indirect effects on emotional exhaustion and intent to leave, with missed nursing care and scope of practice mediating the relationships. More specifically, a better work environment would be negatively associated with missed nursing care, emotional exhaustion, and turnover intentions while positively associated with increased scope of practice.

## Methods

### Design, Sample, and Data Collection

This cross-sectional study was conducted in New Brunswick, a province in eastern Canada, in 2022. Data were collected through a self-reported questionnaire administered online to all hospital-based registered nurses working in the province. The questionnaire took approximately 30 minutes to complete and was accessible online for a 6-week period between May and July 2022. All hospital-based nurses registered with the provincial regulatory body, the Nurses’ Association of New Brunswick (N = 4585), were invited to participate via an online invitation. A reminder email was sent 2 weeks after the initial email. Passive recruitment was also used on social media, by sharing a poster and a short video with the survey link. The survey was completed by 419 nurses, a response rate of 9.1%. We considered our sample size adequate, based on the recommended 10 to 20 participants for each estimated parameter. A minimum sample of 240 would thus be adequate.^51^

The ethics board of the Université de Moncton approved this study (approval number 2122-077) in April 2022. All participants had to read and accept the letter of intent via the survey platform prior to completing the questionnaire. Mental health resources, which were available 24 hours a day, were provided on the consent page and at the end of the questionnaire.

### Measures

The province of New Brunswick has a bilingual nursing workforce; we therefore chose validated English and French surveys, allowing the participants to choose the language they were most comfortable with when responding to the survey. Demographic data collected were gender, age, years of experience as a nurse, employment status, and hospital size.

#### Work environment

The work environment was measured using 5 subscales from the 22-item Nursing Work Index–Extended Organization (NWI-EO).^52^ The NWI-EO is based on the Nursing Work Index–Revised^53^ which is commonly used to measure nurses’ work environment. The following subscales were used: (1) support from senior nurses; (2) staff adequacy; (3) communication on the unit; (4) interruptions in tasks; and (5) shared values concerning care provided. These 5 subscales were chosen because they reflected the organizational components of the work environment which could influence the care given to patients. A 4-point Likert scale ranging from 1 (strongly agree) to 4 (strongly disagree) was used to measure each subscale, with a higher score indicative of a poor organizational work environment. The total NWI-EO scores were used in the analysis. A sample item from the support from senior nurses’ subscale is “In my current job, there is a nurse manager who is a good manager and leader.” The psychometric properties (validity and reliability) were deemed acceptable.^52^ The internal consistency of the scale was 0.81 for the English version and 0.82 for the French version.

#### COVID-19 impact

The impact of COVID-19 on nurses was measured by 7 items.^54^ We converted the original dichotomous scale to a 5-point Likert scale ranging from 1 (never) to 5 (always) with a higher score indicating a greater negative impact on the nurses. An item from the questionnaire was the following: “At what frequency did you experience an intense workload during the COVID-19 pandemic?” In this study, the Cronbach’s alpha coefficient was 0.77 (English version) and 0.80 (French version).

#### Scope of practice

Nurses’ scope of practice was measured by the Actual Scope of Nursing Practice (ASCOP) questionnaire.^33^ This 26-item scale includes 6 subscales: (1) assessment and care planning; (2) teaching; (3) communication and care collaboration; (4) integration and supervision of staff; (5) quality of care provided; and (6) patient safety and knowledge updating and utilization. A 6-point Likert scale ranging from 1 (never) to 6 (always) was used to measure each subscale with a high score indicative of an optimal nursing practice. An item for the communication and care collaboration subscale is “I coordinate the work of the nursing team to meet the needs of the patients and family.” In addition to the subscales, items were divided by 3 complexity levels. Higher complexity items were excluded for this survey, as they represented advanced nursing practice according to the Canadian Nurses Association,^55^ thus bringing the total number of items to 18. In this study, the Cronbach’s alpha coefficient was 0.91 for the English version and 0.88 for the French version.

#### Missed nursing care

Missed nursing care was measured with 2 subscales from part A of the MISSCARE questionnaire^28^: interventions (basic needs) and interventions (individual needs); bringing the total number of items to 12. The subscales for assessment and planning were not used in this study, as the content was similar to the assessment and care planning subscale of the ASCOP. Furthermore, we were concerned that the length of the survey would deter potential participants. A 5-point Likert scale was used, ranging from 1 (never missed) to 5 (always missed), with a higher score indicating a greater prevalence of missed nursing care. Frequency of missing or omitting elements of patient care, such as ambulation, hygiene, and medication administration, was measured. The validity and reliability of the MISSCARE questionnaire have been well documented in the literature.^23^ Cronbach’s alpha coefficients were 0.89 (English version) and 0.84 (French version) in this study.

#### Emotional exhaustion

Emotional exhaustion was measured with an 8-item subscale from the Oldenburg Burnout Inventory scale.^38^ A 4-point Likert scale ranging from 1 (strongly agree) to 4 (strongly disagree) was used, with a high score indicating increased burnout symptoms. A sample item for the emotional exhaustion scale is “During my work, I often feel emotionally drained.” A score of 2.25 or higher on the emotional exhaustion scale is considered high. The entire questionnaire is both reliable and valid.^38^ In this study, Cronbach’s alpha coefficients were 0.89 (English version) and 0.84 (French version).

#### Intent to leave

Intent to leave was measured using a single item from a 2-item scale on whether nurses were considering leaving the nursing profession.^56^ A 5-point Likert scale ranging from 1 (very likely) to 5 (very unlikely) was used. A lower score meant a higher turnover intention. The validity and reliability of the entire scale are well documented.^57^

### Data Analysis

Initial data analysis was performed using IBM SPSS Statistics for Windows, Version 28.0 (Armonk, NY). Descriptive analyses (means, standard deviations, and frequency distributions) were performed on the main variables. An assessment of multicollinearity, skewness, and kurtosis was also done. Cronbach’s alpha was used to evaluate the reliability of the chosen scales. Independent paired *t*-tests were performed to evaluate differences in mean scores between English and French questionnaires. We found no significant differences between the two versions of the questionnaire. The results are shown in the Supplemental Material. The decision was therefore made to merge the data from the English and French questionnaires for the subsequent analyses. Data were then transferred to MPlus^58^ Version 8 (Los Angeles, CA) for mediation analyses. Full information maximum likelihood was used to estimate missing values. Models were estimated using direct and indirect paths from 6 variables. To assess the overall model fit, the following fit indices were used^59^: chi-square value (χ^2^), the Comparative Fit Index (CFI), the Tucker-Lewis Index (TLI), the Standard Root Mean Square Residual, and the Root Mean Square Error of Approximation (RMSEA). The results of the mediation analysis were interpreted by calculating standardized regression estimate scores (β). A bootstrapping procedure of 1000 iterations was used to estimate the 95% confidence intervals.

## Results

A majority of the participants (95%) were female with an average age of 39 years (SD = 11.19). They had an average of 14 years (SD =11.05) of experience, with 70% working full time. The vast majority of nurses worked in either acute care (34%) or intensive care (26%). Finally, 43% of the participants worked in medium-sized hospitals (200-399 beds). Comparison of our sample to the provincial nurse registration data indicated that the final sample closely matched the characteristics (i.e., age, gender) of hospital-based nurses working in the province.

The mean score of the NWI-EO was 2.95 (SD = 0.40), indicating a poor work environment. The COVID-19 pandemic had a great impact on the nurses’ practice, with a mean score of 3.94 (SD = 0.72). In relation to the scope of practice, participants had a mean score of 4.52 (SD = 0.76), indicating a slightly sub-optimal nursing practice. Missed nursing care revealed a mean score of 2.86 (SD = 0.86), indicating that care is often missed. Nurses reported high levels of emotional exhaustion (x̄ = 3.17, SD = 0.53). Finally, 73% of nurses considered leaving the profession (x̄ = 1.99). Table 1 presents the means, standard deviations, and correlations between the variables. Significant positive correlations were found between work environment and emotional exhaustion (*r* = 0.59, *p* < .001) as well as between COVID-19 and emotional exhaustion (*r* = 0.57, *p* < .001). Finally, a significant negative correlation was found between the work environment and the intent to leave the profession (*r* = −0.44, *p* < .001).

**Table 1.** Means, Standard Deviations, and Correlations of Main Variables (N = 419).

	M	SD	1	2	3	4	5	6
Work environment	2.95	0.40	—					
COVID	3.94	0.72	0.52**	—				
Scope of practice	4.53	0.76	−0.20**	−0.08	—			
Missed nursing care	2.86	0.86	0.44**	0.39**	−0.21**	—		
Emotional exhaustion	3.17	0.53	0.59**	0.57**	−0.08	0.31**	—	
Intent to leave	1.99	1.08	−0.44**	−0.30**	−0.01	−0.23**	−0.47**	—
Age	38.71	11.19	−0.19**	−0.34**	−0.03	−0.20**	−0.21**	0.14**

Abbreviation: M: mean; SD: standard deviation.

** p < .001.

**Figure 1.**
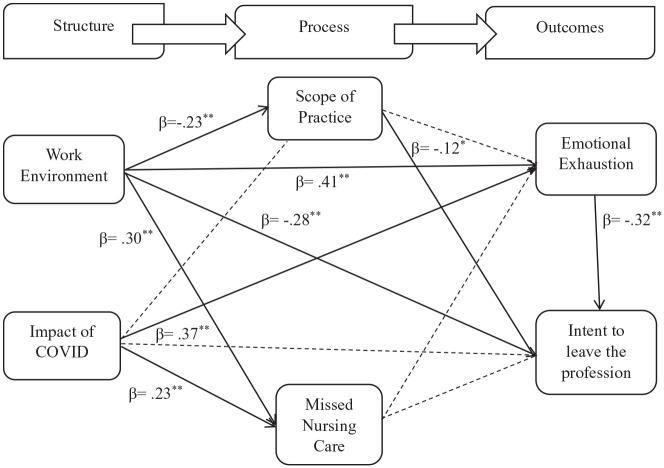
Final model. ---- Indicates non-significant path. *p < .05. **p < .001.

The final model provides a good fit to the data (χ^2^ = 4.371, df = 2, p = .112, CFI = 0.995, TLI = 0.954, RMSEA = 0.055). The model explained 45% of the variance of emotional exhaustion and 28% of the variance of intent to leave. Figure 1 shows the coefficients for each direct path within the 6-factor model. Work environment had a significant positive effect on missed nursing care (β = 0.304, p < .001) and emotional exhaustion (β = 0.410, p < .001), and a significant negative effect on scope of practice (β = −0.229, p < .001) and intent to leave (β = −0.283, p < .001). Thus, a poor work environment is associated with an increase in missed nursing care and emotional exhaustion, accompanied by a limited scope of practice and increased turnover intentions. As well, the COVID-19 pandemic has a significant effect on missed nursing care (β = 0.230, p < .001) and emotional exhaustion (β = 0.367, p < .001), which indicates an increase in missed nursing care and emotional exhaustion. There was also a direct positive significant effect between age and intent to leave (β = 0.110, p = .040), indicating that younger nurses have increased turnover intentions. However, the relationships between the impact of COVID-19 and scope of practice (β = 0.055, p = .331) as well as intent to leave (β = 0.078, p = .159) were not significant.

Table 2 describes the estimates of the indirect paths of our model. Our hypothesized model proposed that missed nursing care and scope of practice would act as mediators. Missed care did not mediate the relationship between work environment and emotional exhaustion or intent to leave. Similarly, missed nursing care did not mediate the relationship between COVID-19 and emotional exhaustion or intent to leave. Our hypotheses regarding missed care as a mediator were therefore rejected. Nonetheless, scope of practice partially mediated the relationship between work environment and intent to leave (work environment → scope of practice → intent to leave; β = 0.026, p = .040, 95% CI [0.007, 0.057]), as the direct effect between work environment and intent to leave remained statistically significant.

**Table 2.** Parameter Estimates of Indirect Paths (N = 419).

Indirect effects	Β	SE	p	95% CI
Work environment → scope of practice → EE	−0.007	0.009	.477	[−0.029, 0.008]
Work environment → missed care → EE	−0.003	0.016	.850	[−0.039, 0.024]
Work environment → scope of practice → TI	0.026	0.013	.040	[0.007, 0.057]
Work environment → EE → TI	−0.130	0.029	.000	[−0.195, −0.083]
Work environment → missed care → TI	−0.012	0.015	.440	[−0.046, 0.014]
Work environment → scope of practice → EE → TI	0.002	0.003	.491	[−0.003, 0.010]
Work environment → missed care → EE → TI	0.001	0.005	.850	[−0.008, 0.013]
COVID → scope of practice → EE	0.002	0.003	.638	[−0.002, 0.013]
COVID → missed care → EE	−0.002	0.012	.847	[−0.028, 0.020]
COVID → scope of practice → TI	−0.006	0.007	.392	[−0.026, 0.004]
COVID → EE → TI	−0.116	0.026	.000	[−0.176, −0.070]
COVID → missed care → TI	−0.009	0.011	.417	[−0.032, 0.013]
COVID → scope of practice → EE → TI	−0.001	0.001	.643	[−0.004, 0.001]
COVID → missed care → EE → TI	0.001	0.004	.850	[−0.006, 0.010]

Abbreviations: SE: standard error; CI: confidence interval; EE: emotional exhaustion; TI: turnover intention.

Emotional exhaustion also partially mediated the relationship between work environment and intent to leave (work environment → emotional exhaustion → intent to leave; β = −0.130, p < .001, 95% CI [−0.195, −0.083]). Finally, emotional exhaustion fully mediated the relationship between the impact of COVID-19 and intent to leave (COVID → emotional exhaustion → intent to leave; β = −0.116, p < .001, 95% CI [−0.176, −0.070]).

## Discussion

The purpose of this study was to evaluate the mediating effect of missed care and scope of practice on the relationship between nurses’ work environment and nurse outcomes (emotional exhaustion and turnover intentions) during the final waves of the pandemic. Our findings show significant direct relationships between the work environment and scope of practice, missed nursing care, emotional exhaustion, and turnover intentions. The impact of the work environment on missed nursing care is similar to multiple recent studies.^12,24,25^ Nonetheless, missed nursing care did not predict turnover intentions, which contradicts other research.^6,23,25^ Missed care also did not mediate the relationships between the work environment on either emotional exhaustion or intent to leave, similar to a recent mediation analysis by Liu et al.^12^ Labrague et al^19^ and Alfuqaha et al^60^ have shown that the prevalence of missed care has increased during the pandemic, mainly because of inadequate nurse staffing. Although our study focuses on the later waves of the pandemic, our findings indicate that care is often missed. The deterioration of the work environment, caused by successive waves of the pandemic, may have caused a generalized acceptance that missed nursing care is now the norm, given the limited resources and ongoing prioritizing of patient care. As most studies took place prior to the pandemic, the impact of missed care on nurse outcomes (i.e., turnover intentions) may also have changed.

Our study provides new knowledge on scope of practice and nurse outcomes. There are many organizational barriers to scope of practice enactment, such as time constraints and lack of available resources to perform tasks that could be delegated.^35,36^ We found that scope of practice partially mediated the relationship between work environment and turnover intentions. While the enactment of nurses’ scope of practice has not been vastly studied, our findings suggest that it plays a more important role in turnover intentions than missed nursing care. The inability to provide nursing care that is congruent with one’s education and professional values could increase nurses’ cognitive dissonance and consequently lead to moral distress.^61^ Constraints within the health care system^61,62^ may be forcing nurses to make decisions which go against their values, thus increasing the risk of moral distress, which is known to increase burnout symptoms^21,62^ and turnover intentions.^21^ While there are many studies regarding the moral injuries that occurred among nurses during the pandemic,^17,21,44,62^ there needs to be additional research examining the complex relationship between work environment, scope of practice, moral distress, and turnover intentions.

This study highlights the important role burnout symptoms play in predicting turnover intentions and contributes to the existing literature on healthy work environments and nurse outcomes.^4,11,37,39,42,46^ Our findings show that emotional exh-austion partially mediated the relationship between work environment and turnover intentions. Its impact on nurses’ well-being cannot be overstated, with 77% of nurses in Canada reporting a decline in their mental health.^63^ To mitigate burnout, it is important to empower nurses, by listening to their concerns and by allowing their participation in unit-based and hospital decisions.^4,11,42,45^ Organizations tend to view burnout as an individual issue, consequently targeting interventions at the individual level, such as encouraging nurse resiliency and mindfulness to cope with workplace stressors.^39,44^ However, there is now enough empirical evidence indicating that burnout in nursing should be addressed as an organizational phenomenon.^37^ As such, mitigating burnout symptoms must focus on improving nurses’ work environment.^11,39,43-45^ Ultimately, high levels of burnout in the nursing workforce have an enormous impact not only on nurses’ well-being, but on patients and the entire health care system.

Finally, we found that emotional exhaustion completely mediated the relationship between COVID-19 impact and intent to leave, adding to the empirical evidence of the pandemic’s influence on turnover intentions.^4,42,43,48,57^ Prior to the pandemic, predictors of turnover intentions included organizational commitment, job satisfaction, and work environment.^64^ These existing predictors have been exacerbated by the de-mands of the pandemic^45,48^ as well as the development of new stressors related to fear of disease, anxiety, and stress.^48^ We also found that younger nurses are more likely to consider leaving, in line with other studies.^4,11,25,39,48,64^ Several strategies could encourage young nurses to remain, such as an improvement in work-life balance (e.g., family-friendly working hours, childcare, flexible scheduling), opportunities for professional dev-elopment,^4^ and a prolonged mentorship program for nurses entering the profession.^43,45^ With an increased number of nurses approaching retirement, several changes can also be made to ensure their retention, such as an adjustment of work circumstances (e.g., fewer night shifts, less demanding physical work)^4^ and flexible scheduling.^43^ To this end, there are a growing number of national and international reports identifying evidence-based retention strategies.^43-45^

There are several limitations to this study. First, the cross-sectional design does not provide the ability to prove causation, nor evaluate how the outcomes may evolve over time. There is an urgent need for similar longitudinal studies examining these variables over a period of years. Second, the use of self-report measures can also contribute to biased responses. Third, the length of the questionnaire may have discouraged some participants to complete the survey. We had a response rate of 9%, which can limit the generalizability of our results. Furthermore, the participants in our study were slightly younger (mean age of 39), than all hospital-based nurses in the province (mean age of 44), indicating that younger nurses were more represented in our sample.^65^ The online nature of our survey may have therefore caused a selection bias. Finally, our results would need to be explored with more objective measures, such as the internal turnover within the hospital or rate of absenteeism, which would have strengthened this research.

In the context of the global nursing shortage, it is urgent to retain nurses in the health care system to provide quality care, by allowing them workplace autonomy and ensuring their inclusion in decision-making.^43-45,48^ Allowing nurses to function to their full scope of practice and ensuring a healthy work environment could promote the well-being of nursing staff and greatly improve patient, nurse, and organizational outcomes. However, effective strategies such as workforce planning and ensuring adequate resources must be put in place to ensure the improvement of nurses’ work environment and to reduce turnover intentions. Unfortunately, governmental actions are often focused on recruitment^45^ and do not address the pressing need for a transformation of nurses’ work environment. While this approach may increase the number of nurses in the short term, keeping the work environment as is will only continue to increase turnover. Decision-makers need to shift their focus on reducing organizational constraints, such as inadequate staffing levels and precarious work conditions, to ensure nurse retention.

Even though our study took place during the later waves of the pandemic, we found that a high percentage of nurses in New Brunswick had burnout symptoms and were considering leaving the profession. This is extremely troubling. The multiple direct and indirect pathways leading to turnover intentions underline the complex interactions among these variables. Although nurses’ work environment has always been challenging, the pandemic appears to have had a long-term impact on nurses, which is exacerbated by the severe nursing shortage. Even though there are numerous reports on strategies to improve the work environment, there are ongoing barriers to implementing targeted strategies at the organizational level. Our research highlights the urgency to implement changes within our health care system, notably by improving the work environment and allowing nurses to enact their full scope of practice, ensuring their retention.

## Supplemental Material

sj-pdf-1-wjn-10.1177_01939459241230369 – Supplemental material for Impact of the Work Environment on Nurse Outcomes: A Mediation Analysis Supplemental material, sj-pdf-1-wjn-10.1177_01939459241230369 for Impact of the Work Environment on Nurse Outcomes: A Mediation Analysis by Caroline Boudreau and Ann Rhéaume in Western Journal of Nursing Research
