# Supplementary material for: Impact of the Work Environment on Nurse Outcomes: A Mediation Analysis
Source: West J Nurs Res. 2024 Feb 11;46(3):210–8. doi: 10.1177/01939459241230369 (PMC10903131; doi:10.1177/01939459241230369)
Supplement: sj-pdf-1-wjn-10.1177_01939459241230369 – Supplemental material for Impact of the Work Environment on Nurse Outcomes: A Mediation Analysis [file sj-pdf-1-wjn-10.1177_01939459241230369.pdf]

Supplementary Table. *t*-tests results comparing English and French questionnaires

	English	French		
**Variables**	Mean (SD)	Mean (SD)	*t*-value (df)	*p* value
Work environment	2.97 (.41)	2.92 (.38)	1.03(417)	.306
COVID-19	3.93 (.63)	3.99 (.93)	-.59 (137)	.554
Missed care	2.85 (.87)	2.92 (.83)	-.69 (407)	.494
Scope of practice	4.55 (.77)	4.47 (.74)	.98 (417)	.372
Emotional exhaustion	3.18 (.50)	3.09 (.61)	1.60 (153)	.110
Intent to leave	2.02 (1.16)	1.92 (.76)	.93 (277)	.352

Note. SD = standard deviation, df = degrees of freedom
